# Impact of social determinants of health on the outcomes of Latin American children with Multisystem Inflammatory Syndrome (MIS‐C)

**DOI:** 10.1002/ppul.27313

**Published:** 2024-12-26

**Authors:** Danilo Buonsenso, Anna Camporesi, Charles Sawaya, Rolando Ulloa‐Gutierrez, Enrique Faugier‐Fuentes, Lourdes Dueñas, Rolando A. Paternina‐de la Ossa, Beatriz A. Llamas‐Guillén, Luisa B. Gámez‐González, Nancy Gálvez‐Rafael, Andrea Gatica, Patricia Saltigeral‐Simental, Adán Cuatecontzi‐Romero, Flávia Jacqueline Almeida, Shirley Cuan, Elmer H. Zapata‐Yarlequé, Sandra Beltrán, Erika Reina‐Bautista, Adrián Collia, Gabriela Ivankovich‐Escoto, Jaime Fernández‐Sarmiento, Adriana H. Tremoulet, The REKAMLATINA‐3 MIS‐C Study Group Investigators, Elizabeth Moreno, Jimena García‐Silva, Issa L. López‐Medina, Manuel Munaico, Róger Hernández, Gian Huamán, Maria C. Cervi, Alejandro Ellis, Daniel Jarovsky, Lorena Franco, Kathia Luciani, Enrique Chacon‐Cruz, Alejandro Díaz, Maynor G. Bravo‐López, Marco A. Yamazaki‐Nakashimada, Itzel Ríos‐Olivares, Martha Márquez‐Aguirre, Adriana Yock‐Corrales, Mónica Pujadas, Ana V. Villarreal‐Treviño

**Affiliations:** ^1^ Area Pediatrica, Dipartimento di Scienze della Vita e Sanità Pubblica Università Cattolica del Sacro Cuore Rome Italy; ^2^ Department of Woman and Child Health and Public Health Fondazione Policlinico Universitario A. Gemelli Rome Italy; ^3^ Pediatric Anesthesia and Intensive Care, Vittore Buzzi Children's Hospital Milano Italy; ^4^ University California San Diego San Diego California USA; ^5^ Servicio de Infectología Pediátrica, Hospital Nacional de Niños “Dr. Carlos Sáenz Herrera”, Caja Costarricense de Seguro Social (CCSS) San José Costa Rica; ^6^ Universidad de Ciencias Médicas (UCIMED) e Instituto de Investigación en Ciencias Médicas (IICIMED) San José Costa Rica; ^7^ Academia Nacional de Medicina de Costa Rica (ACANAMED) San José Costa Rica; ^8^ Servicio de Reumatología Hospital Infantil de México Federico Gómez Ciudad de México México; ^9^ Servicio de Infectología Hospital Nacional de Niños Benjamín Bloom San Salvador El Salvador; ^10^ Servicio de Infectología Universidad de Sāo Paolo Sao Paolo Brazil; ^11^ Servicio de Alergología e Inmunología, Hospital del Niño Morelense Cuernavaca; Morelos México; ^12^ Departamento de Inmunología Hospital Infantil de Especialidades de Chihuahua Chihuahua México; ^13^ Servicio de Infectología Hospital Roosevelt Ciudad Guatemala Guatemala; ^14^ Servicio de Infectología Hospital Juan Pablo II Ciudad Guatemala Guatemala; ^15^ Servicio de Infectología Star Médica Hospital Infantil Privado Ciudad de México México; ^16^ Servicio de Reumatología Hospital de la Niñez Oaxaqueña “Dr. Guillermo Zárate Mijangos” Oaxaca México; ^17^ Servicio de Infectología Santa Casa de São Paulo School of Medical Sciences São Paulo Brazil; ^18^ Servicio de Pediatría Hospital Herrera Llerandi Ciudad de Guatemala Guatemala; ^19^ Zona Pediátrica Hospital de Niños Ciudad de Guatemala Guatemala; ^20^ Servicio de Pediatría Clínica San Felipe Lima Peru; ^21^ Servicio de Infectología Clínica Pediátrica Colsanitas Bogotá Colombia; ^22^ Servicio de Infectología Hospital Regional de Alta Especialidad Ixtapaluca Ixtapaluca México; ^23^ Servicio de Cardiología Sanatorio Mater Dei Buenos Aires Argentina; ^24^ Servicio de Inmunología y Reumatología Hospital Nacional de Niños “Dr. Carlos Sáenz Herrera”, Caja Costarricense de Seguro Social (CCSS) San José Costa Rica; ^25^ Department of Pediatrics and Intensive Care, Fundación Cardioinfantil‐Instituto de Cardiología Universidad de La Sabana Bogotá Colombia; ^26^ Department of Pediatrics & Kawasaki Disease Research Center University of California San Diego (UCSD) & Rady Children's Hospital San Diego California USA; ^27^ University of California San Diego California USA; ^28^ Facultad de Medicina, Universidad Autónoma de Nuevo León Monterrey; ^29^ Clínica Pediátrica Colsanitas Bogotá Colombia; ^30^ Clínica San Felipe Lima Peru; ^31^ Universidade de Sāo Paolo Sāo Paolo Brazil; ^32^ Sanatorio Mater Dei Buenos Aires Argentina; ^33^ Santa Casa de Sāo Paulo School of Medicinal Sciences Sāo Paulo Brazil; ^34^ Hospital Infantil Municipal de Córdoba Córdoba Argentina; ^35^ Hospital de Especialidades Pediátricas Omar Torrijos Herrera Ciudad de Panama Panama; ^36^ Hospital General de Tijuana; Tijuana, México & Think Vaccines LLC Houston Texas USA; ^37^ Hospital General de Medellín Medellín Colombia; ^38^ Hospital Herrera Llerandi, Ciudad Guatemala Guatemala; ^39^ Instituto Nacional de Pediatría; Ciudad de México México; ^40^ Hospital Nacional de Niños “Dr. Carlos Sáenz Herrera”, Centro de Ciencias Médicas, Caja Costarricense de Seguro Social (CCSS) San José Costa Rica; ^41^ Hospital Pediátrico Centro Hospitalario Pereira Rossell Montevideo Uruguay; ^42^ Hospital Regional Materno Infantil de Alta Especialidad Monterrey México

**Keywords:** children, Latin America, multisystem inflammatory syndrome, risk factors, social determinants of health

## Abstract

**Importance:**

There is growing understanding that Social Determinants of Health (SDH) impact on the outcomes of different pediatric conditions. We aimed to determine whether SDH affect the severity of MIS‐C.

**Design:**

Retrospective cohort study, 2021–2023. Children and adolescents with MIS‐C younger than 18 years of age fulfilling the MIS‐C CDC definition within the REKAMLATINA network were invited to participate. We assessed the **i**mpact of SDH on the risk of children with MIS‐C to be diagnosed with shock, need of inotropes, respiratory support, transfusion, and death.

**Results:**

Two hundred and seventy seven patients from 30 centers in 13 countries were included. Of them, 241 children from the four most‐represented countries were included in the final analysis. Food insecurity, higher distance from a health center, not possessing a private vehicle to transport the patient to hospital, and having a home in poor condition, were associated with low LVEF, need of transfusion, shock, and need for respiratory support, when controlling for age, BMI, and ethnicity. The Score of Social Disadvantage was associated with Shock (OR: 1.35, P: 0.011, 95% CI: 1.07–1.71), Respiratory support (OR: 1.39, P: 0.005, 95% CI: 1.1–1.75), Transfusion (OR: 1.63, P0.013, 95% CI 1.1–2.41), but not death (OR: 0.76, P: 0.38, 95% CI: 0.41–1.40).

**Conclusions:**

Among a large cohort of Latin American children with MIS‐C, SDH negatively affect outcomes. These findings reinforce the need for better investigation of the role of SDH in MIS‐C and other inflammatory conditions and may guide public health interventions.

## 1 INTRODUCTION

Multisystem Inflammatory Syndrome (MIS‐C) is the most severe complication of SARS‐CoV‐2 infection in children,^1^ being an inflammatory condition with systemic involvement that frequently requires organ support in pediatric intensive care units and, sometimes, is associated with fatal outcomes.^1^

MIS‐C is uniformly recognized as a hyperinflammatory immune response triggered by SARS‐CoV‐2 infection,^1^, ^2^ and therefore treated with immunomodulatory agents. Several studies have demonstrated that intravenous immunoglobulins (IVIG), steroids, and biological agents are effective in successfully treating these children.^3^, ^4^ That said, an evaluation of risk factors, including Social Determinants of Health (SDH), that would warrant more aggressive initial therapy, has not been done.

So far, non‐Hispanic black and Latino children have been shown to have an increased prevalence of MIS‐C compared to white children.^5^ In addition, MIS‐C cases in developing countries have shown higher mortality as compared to high‐income countries, which suggest a role for SDH in the severity of MIS‐C.^5^, ^6^ These observations have led clinicians to speculate that environmental and/or social factors may play a role in defining a different risk stratification for MIS‐C. Although the incidence of MIS‐C following SARS‐CoV‐2 infection has significantly declined,^7^ understanding the impact of environmental/social factors on the outcomes of MIS‐C remains still relevant as identifying these factors could help reduce health disparities overall. In addition, although MIS‐C and Kawasaki disease (KD) are immunologically different conditions,^2^ they still share several similarities and similar responses to the same treatments, suggesting that if we understand the impact of environmental/social factors on MIS‐C outcomes, we may be able to better understand KD, the most common cause of acquired heart disease in children throughout most of the world.^8^

Thus, the aim of the present study was to investigate if SDH had an impact on severity of MIS‐C disease in a large cohort of Latin American children.

## 2 METHODS

REKAMLATINA is a multinational, multicenter research network (initially established for Kawasaki disease but then expanded to include patients with MIS‐C) of the main pediatric and general referral hospitals in Latin America that maintains a detailed observational registry of KD cases and, since the COVID‐19 pandemic, MIS‐C, from 64 participating centers across 16 Latin American countries: Argentina, Bolivia, Brazil, Chile, Colombia, Costa Rica, Cuba, Dominican Republic, Ecuador, El Salvador, Guatemala, Honduras, Mexico, Panama, Paraguay, Peru, Puerto Rico, Uruguay, and Venezuela.^9^, ^10^ The REKAMLATINA REDCap database has received institutional review board approval at the University of California, San Diego (La Jolla, CA, USA), and the study was approved at each individual institution enrolling subjects in the REKAMLATINA MIS‐C REDCap database. Subjects were eligible for inclusion in the registry if they were diagnosed with MIS‐C based on the CDC definition.^11^

The original database, launched for MIS‐C in early 2020, included data on demographics, previous diseases, clinical signs and symptoms, vital parameters upon admission to the hospital, laboratory as well as radiological exams, echocardiographic findings, destination of the patient (PICU or ward), kind of support needed and duration of support, therapies, complications, and outcome. Further details on how data were collected in the data set are provided in previous REKAMLATINA studies.^12^

Starting in early 2021, the database also collected data on social and economic aspects of the family and patient. The collected information is presented in Table 1.

**Table 1:** Collected social and economic data.

Social and economic aspects	
Family structure	Mother and father
	Only mother
	Only father
	Other
Age of caregivers	
Education of caregivers	Not gone to school
	Primary school not completed
	Primary school completed
	Secondary school not completed
	Secondary school completed
	Technical/professional school
	University
Characteristics of the household	Number of people living together
	Presence of people with disabilities
	Presence of children with disabilities
	Number of children under 5 years, between 5 and 11 years and over 11 years of age
	Number of rooms in the household
Facilities available in the household	Electricity
	Running water
	Well water
	In‐house toilet
	Latrine
	Collection of solid waste
Food insecurity	
Distance from healthcare center	
Mean of transportation to healthcare center	Walking
	Cart
	Bicycle
	Motorcycle
	Public transport
	Taxi
	Private vehicle
Household income	
Means of payment of hospital admission	Public insurance
	Private insurance
	Savings
	Selling properties
	Loan
	Other

Among the different social determinants, we then selected five because of their stronger and recurrent associations with important clinical outcomes as suggested by previous literature^13^: maternal educational level, inadequate nutrition, household income, distance from the closest health center and means of transportation to the hospital. The five items were summed to create a Score of Social Disadvantage.

The main hypothesis of this study was that SDH can impact the outcomes of MIS‐C. Since the most severe forms of MIS‐C are characterized by left ventricular dysfunction, the primary outcome was to assess the role of SDH on left ventricular dysfunction, measured by worst left ventricular ejection fraction during hospital stay. Secondary outcomes were to evaluate the role of SDH on other clinically relevant outcomes, including receiving a diagnosis of shock, the need for respiratory support, transfusion and death and the correlation between the Score of Social Disadvantage and clinical outcomes.

### 2.1 Statistical analysis

Quantitative variables were described by median and interquartile range (IQR). Frequencies and percentages were used for qualitative variables. Associations between categorical variables was studied with Pearson's chi‐square test or Fisher's exact test as appropriate.

Since the primary outcome was to assess the role of SDH on the severity of heart involvement in the cohort, the worst Left Ventricular Ejection Fraction (LVEF, %) during hospital stay was studied as the outcome of a multivariable linear regression model including each of 8 SDH (maternal education; household income; living in a house in good state; food insecurity; home overcrowding determined by the number of people per room of the house; distance from the hospital; means of transportation to the hospital; kind of payment for hospital) and other covariates that are known in the literature to affect the outcomes of SARS‐CoV‐2 infection in both adults and children and MIS‐C (age, BMI and ethnicity).^14^, ^15^ Multilevel mixed‐effects models were also used to study the need for respiratory support, including a random effect to handle the data variability between the single centers. Results were reported as odds ratios (ORs) and 95% confidence intervals (CI). The Intraclass Correlation (ICC) of the models was also described.

**Figure 1:**
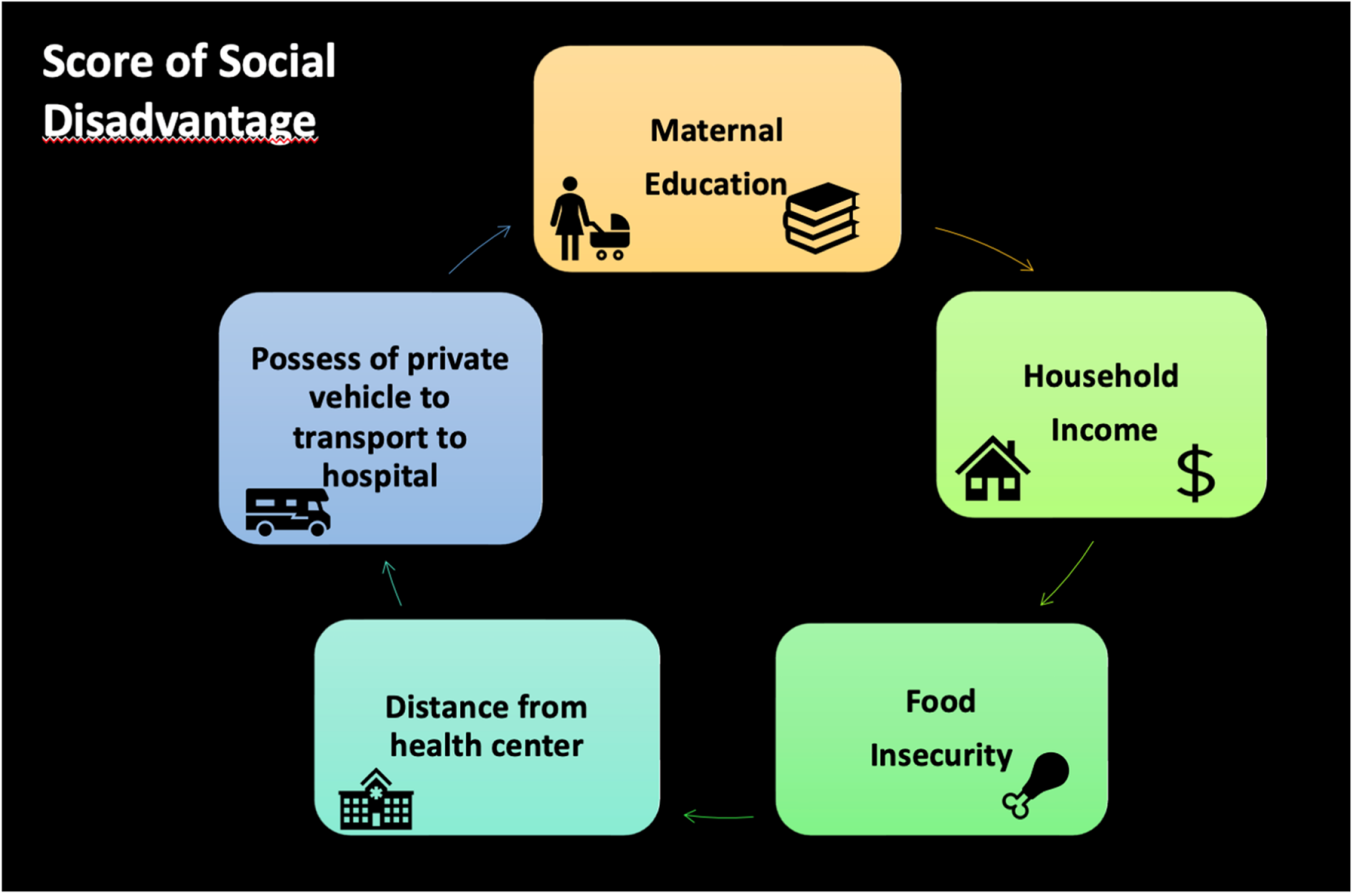
Items included in the Score of Social Disadvantage. [Color figure can be viewed at wileyonlinelibrary.com]

The Score of Social Disadvantage was built considering 5 SDH variables that have been previously shown to be associated with increased severity of MIS‐C (maternal educational level, inadequate nutrition, household income, distance from the closest health center and means of transportation to the hospital, see Figure 1) and categorizing each into a binary variable: maternal education lower than completed high school (1 point)/superior to completed high school; (0 points) history of any skipped meals (1 point)/no skipped meals in the last 30 days; (0 points) income lower than the rest of the country (1 point)/higher than the rest of the country (0 points); distance higher than 1 h from hospital (1 point); lower than 1 h from hospital (0 points)/transportation of the patient to hospital with any other mean than private vehicle (1 point)/with a private vehicle (0 points). The score could therefore range from 0 to 5 points with higher scores in more disadvantaged situations. The score was tested with all the above‐mentioned clinical outcomes with logistic or linear regression as appropriate.

All statistical tests were two‐sided and the level of statistical significance was set at 0.05. Data have been analyzed with Stata 18 BE (StataCorp. 2023. Stata Statistical Software: Release 18. College Station, TX: StataCorp LLC.).

## 3 RESULTS

### 3.1 Study population

**Figure 2:**
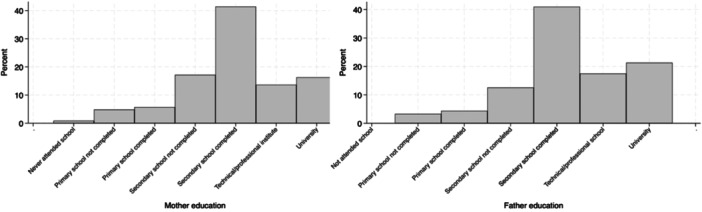
Representation of education of the mother and father in the study cohort.

**Figure 3:**
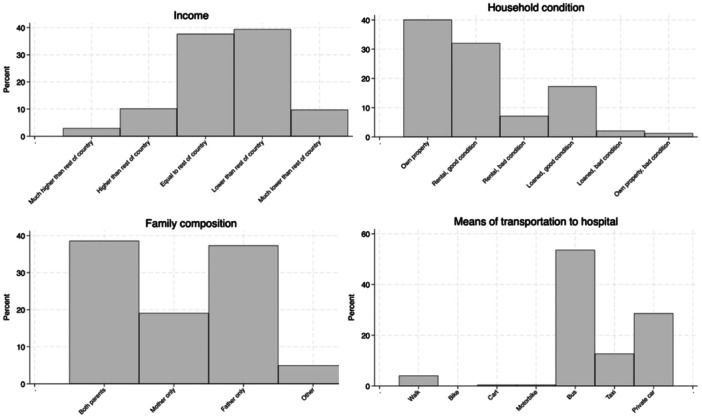
Representation of family composition, household condition, income and means of transport to hospital in the study cohort.

One thousand five hundred and thirty nine patients from 16 countries were included in the original database. Cases without completed social determinants data were excluded, leaving 277 patients from 30 centers in 13 countries for analysis. Details about the main demographic and clinical characteristics of the children enrolled are described in Table 2, and details about the SDH of the cohort are described in Figures 2 and 3. Nationality of participants was: Bolivia 1 (0.4%); Brazil 29 (10.5%); Chile 4 (1.4%); Colombia 8 (2.9%); Costa Rica 3 (1.1%); Cuba 1 (0.4%); Guatemala 38 (13.7%); Mexico 144 (52%); Panama 3 (1.1%); Peru 7 (2.5%); El Salvador 30 (10.8%); Uruguay 1 (0.4%). Four countries (Mexico, Guatemala, El Salvador and Brazil) contributed to the majority of patients (241/277) while the others contributed with 1–8 patients which was therefore considered not significant to be representative. In the final analysis, thus, we considered only the patients from the 4 abovementioned countries.

**Table 2:** Demographic/clinical data of the cohort and symptoms at admission to ED.

Demographic data	Total *N* = 277	Total from the 4 most represented countries *N* = 241
Male sex, % (N)	147 (53.2%)	123 (51.0%)
Age, median (IQR), months	80 (25; 120)	75.0 (30.0–120.0)
Weight, median(IQR), kg	21.5 (13; 36)	21.3 (13.0–35.6)
Height, median(IQR), cm	114 (88; 139)	113.5 (90.0–138.5)
BMI, median (IQR), kg/(m2)	16.8 (15.11; 19.82)	16.7 (15.1–19.8)
Illness day at hospitalization, days	5 (4; 7)	6.0 (4.0–7.0)
Hospital stay, days	6 (5; 9)	6.0 (4.0–9.0)
Race		
White	55 (19.9%)	40 (16.6%)
Afroamerican	5 (1.8%)	4 (1.7%)
Asian	0 (0%)	0 (0.0%)
Indigenous	3 (1.1%)	3 (1.2%)
Mestizo	207 (74.7%)	195 (80.9%)
Missing	7 (2.5%)	0 (0%)
Medical history		
Previously healthy	246 (89.1%)	215 (89.2%)
Heart disease	6 (2.1%)	12 (5.0%)
Blood disease/oncologic diseases	4 (1.4%)	5 (2.1%)
Lung disease	7 (2.5%)	4 (1.7%)
Rheumatological disease	1 (0.4%)	4 (1.7%)
Immunodeficiency	0	1 (0.4%)
Other comorbidities	17 (6.1%)	0 (0.0%)
Number of previous medical consults in the 30 days before		
0	39 (14.1%)	33 (13.8%)
1	86 (31.0%)	73 (30.5%)
2	92 (33.2%)	82 (34.3%)
3	39 (14.1%)	36 (15.1%)
4	13 (4.7%)	11 (4.6%)
5	4 (1.4%)	4 (1.7%)
Missing	4 (1.4%)	
Previous antibiotic therapy	158/274 (57.6%)	145 (60.4%)
Main laboratory parameters		
Hemoglobin Z score	−1.9 (−3.2; −0.79)	−1.9 (−3.1–0.8)
WBC, n/mm^3	11225 (8000; 16350)	11000.0 (7900.0–16200.0)
Neutrophils, %	74 (56; 83)	0.7 (0.6–0.8)
ESR, mm/hr	31.5 (19.0–48.5)	30.0 (18.0–44.0)
CRP, mg/L	103.75 (52.4; 215.5)	96 (49–210.3)
Albumin, g/dL	3.0 (2.6; 3.7)	3.0 (2.6–3.6)
GGT, IU/L	52.0 (24.0–116.0)	52.0 (24.0–115.0)
ALT, IU/L	43.8 (23.6; 83)	45.5 (24.0–82.0)
Ferritin, ng/mL	459.4 (182.0; 923.5)	423.7 (165.3–956.0)

*Note*: Illness Day 1 was considered as first day of fever. Data are reported as N (%) for categorical variables or median (IQR) in case of continuous variables.

Abbreviations: ALT, Alanine Aminotransferase; BMI, Body Mass Index; CRP, C Reactive Protein; ESR, Erythrocyte Sedimentation rate; GGT, Gamma‐glutamil‐transpeptydase.

### 3.2 Social determinants

Social determinants, including parental education level, household conditions and facilities, number of persons living in the household and relative characteristics, means of transportation to the hospital and means of payment to the hospital, are all presented in File S1 and depicted in Figures 2 and 3.

### 3.3 Clinical presentation and need for support

At admission, the median duration of fever before Emergency Department (ED) presentation was 6 (4–7) days. 112 patients (46.5%) presented with dehydration, 70 (29.0%) with cough, 90 (37.6%) with abdominal pain and 88 (36.7%) with shock.

Median Ejection Fraction (EF) at admission was 63 (IQR 58%–69%); 17.4% (42) had an EF lower than 55%. Table 3 describes the support needed and the outcome of the study population.

**Table 3:** Need for support and outcomes in the cohort.

Support/outcomes	Total *N* = 241
IV fluids	161 (66.8%)
Respiratory support, N (%)	114 (47.3%)
Admission to PICU, N (%)	85 (35.2%)
PICU LOS, days median (IQR)	4 (2; 6)
Transfusion, N (%)	26 (10.8%)
Mechanical ventilation, N (%)	38 (15.7%)
Mechanical ventilation duration, days	2 (1–5)
Inotropic support, N (%)	77 (31.9%)
Inotrope duration, days	2 (1; 4)
Acute kidney injury N (%)	17 (7.0%)
Death, N (%)	8 (3.3%)

Abbreviations: IV, Intravenous; LOS, length of stay; PICU, pediatric intensive care unit.

### 3.4 Associations between clinical data and SDH

#### 3.4.1 Ejection fraction

In the multivariable regression analysis for the worst LVEF% documented during admission, food insecurity, distance from the hospital, not possessing a private vehicle to transport the patient to the hospital, having a home in poor condition, were all associated with low EF (Table 4). The analysis was adjusted for age, BMI, and ethnicity.

**Table 4:** Results of multivariable linear regression conducted on worst LVEF%.

Worst LVEF (%)	Coefficient	P > t	[95% conf. interval]	
Distance from hospital	−3.98	0.006	−6.83	−1.13
Not possessing a private vehicle to transport the patient to hospital	−3.62	0.022	−6.72	−0.53
Home in bad condition	−4.39	0.007	−7.54	−1.23

*Note:* Including the nation in the model showed a significantly worse EF% for El Salvador (−6.3; 95% CI: −11.7; −0.83; *p* = 0.024). Variables are adjusted for age, BMI and ethnicity.

Abbreviations: BMI, Body Mass Index; LVEF, Left Ventricular Ejection Fraction.

### 3.5 Respiratory support

The need for respiratory support (which included low flow oxygen and respiratory support of any kind, including invasive ventilation) was correlated with worst LVEF% in bivariate analysis (OR 0.93; 95% CI: 0.90 ‐0.96; *p* < 0.001), as expected. However, not all patients with low LVEF% (in the first two quartiles for instance) received respiratory support (Table 5). Therefore, to assess the potential effect of the center on the use of respiratory support, a multilevel mixed‐effects logistic regression with random effect on the center was conducted on respiratory support and each of the SDH, which showed an IntraClass Correlation between 0.14 and 0.15. The model was also adjusted for having the first PCR (Polymerase Chain Reaction) positive for COVID‐19, to account for a potential pulmonary involvement if still actively infected. Higher distance from the hospital, not possessing a private vehicle to transport the patient to hospital, and having a home in bad condition, were all associated with the need for respiratory support (Table 6). Higher maternal education was a protective factor on need for respiratory support (OR: 0.73; 95% CI: 0.59–0.90; *p* = 0.004), and so was household income (OR: 0.58; 95% CI: 0.42–0.79; *p* = 0.001).

**Table 5:** Association between worst LVEF% quantiles and need for respiratory support.

Worst LVEF (quantiles)	No respiratory support	Respiratory support	Total
<58%	16	41	57
59%–62%	25	24	49
63%–68%	31	20	51
>68%	55	29	84
Total	127	114	241

*Note*: Pearsons' chi‐squared: 20.76; *p* < 0.001.

Abbreviation: LVEF, Left Ventricular Ejection Fraction.

**Table 6:** Risk factors for need for respiratory support among SDH.

Respiratory support	OR	P > t	[95% conf. interval]	
Not possessing a private vehicle to transport the patient to hospital	2.13	0.044	1.00	4.55
Home in bad condition	2.34	0.005	1.29	4.26
Distance from hospital >1 h	3.30	0.029	1.63	6.68

Abbreviation: SDH, Social Determinants of Health.

### 3.6 Transfusion

Maternal education and household income had a protective effect on transfusion (OR for maternal education: 0.68; 95%CI: 0.50–0.92; *p* = 0.013; OR for household income: 0.49; 95%CI: 0.29–0.82; *p* = 0.007).

### 3.7 Score of social disadvantage

A score of social disadvantage was built considering 5 items among SDH (see Figure 1) and tested for correlation with clinical outcomes adjusting it for the nation of patients. It significantly correlated with worst LVEF%, with presence of shock at admission, need of transfusion and the need for respiratory support. It did not correlate with death, likely given the low number of children who died from MIS‐C in this cohort (Table 7).

**Table 7:** Correlation between Score of Social Disadvantage and the clinical outcomes, adjusted for nation.

Outcomes	OR	P > z	[95% conf.]	Interval
Shock	1.35	0.011	1.07	1.71
Respiratory support	1.39	0.005	1.10	1.75
Transfusion	1.63	0.013	1.10	2.41
Death	0.76	0.381	0.41	1.40

The role of nation was also significant in the models: ORs for shock were higher for El Salvador, Mexico, Brazil compared to Guatemala, for respiratory support were higher for El Salvador (OR 9.9, 95% CI: 2.56–38.8; *p* = 0.001), for transfusion were lower for Mexico (OR 0.17; 95% CI: 0.04; 0.63; *p* = 0.008).

## 4 DISCUSSION

To our knowledge, this is the largest study investigating the role of SDH on outcomes of MIS‐C. We found that several SDH, and in particular food insecurity, distance from a health center, not possessing a private vehicle to transport the patient to hospital, payment by other means, and having a home in poor condition, were associated with low LVEF, need of transfusion, shock, and need for respiratory support, when controlling for age, BMI and ethnicity. These findings reinforce a recent, but growing body of literature showing that social factors, from economic status to transportation to household as well as ethnicity and race, are associated with worse outcomes in several major diseases in children.^16^, ^17^

Two manuscripts have previously evaluated SDH in MIS‐C in the US, although in a less comprehensive manner compared with our assessment. Javalkar et al found, in a multicenter retrospective case‐control study, that compared with children with SARS‐CoV‐2 alone, those that also developed MIS‐C were in the lowest socioeconomic status quartile (odds ratio 2.2 [95% confidence interval 1.1–4.4]), highest social vulnerability index quartile (odds ratio 2.8 [95% confidence interval 1.5–5.1]), and were part of racial and/or ethnic minority background.^13^ In a larger cohort of 206 MIS‐C children admitted to Texas Children's Hospital, non‐Hispanic Black patients, and those with increased “Texas area deprivation index” had an increased risk of severe MIS‐C,^18^ defined as having received vasoactive‐inotropic support and/or mechanical ventilation during their hospitalization. These findings are consistent with those in our study, where lower housing quality, food insecurity, educational level and household income were all associated with a more severe form of MIS‐C. Our study reinforced these results in a population outside of the US, emphasizing that SDH can impact medical outcomes on a global scale.

As compared to previous studies, we looked at the need for respiratory support separately, as this may be influenced by local practice (e.g. different thresholds to provide oxygen support). In fact, significant variations in invasive respiratory support in different countries have been documented.^19^ From a pathophysiologic perspective, respiratory support may be needed for pulmonary edema due to poor cardiac function. Our cohort showed a correlation between LVEF and need for respiratory support, as expected, although not all patients with low LVEF received respiratory support. Interestingly, the same SDH factors associated with shock also correlated with respiratory support. In addition, the need for transfusion significantly correlated with SDH factors, specifically higher maternal education and higher household income had a protective effect on it. This finding may be associated with previous evidence that hemoglobin levels may be associated with various dietary and non‐dietary influences originating from household and maternal social factors,^20^ allowing us to speculate that children exposed to more fragile SDH might have had lower pre‐illness hemoglobin levels, leading to higher need for transfusion.

There is increasing awareness that while social factors are linked with worse outcomes in several common pediatric disorders,^21^ SDH are rarely collected systematically in pediatric studies. A recent study showed that European pediatric journals did not commonly report either GEAR (geography, ethnicity, ancestry, and race or religion) or SDH, and there was wide variation in how data were collected and reported.^22^ The lack of routine inclusion of SDH is most probably due to the difficulties in routinely collecting such information. Therefore, such a rigorous assessment in our study, particularly considering the evidence of their significant impact on outcomes, is a strength of our research and reinforces the need to routinely include SDH in pediatric studies, calling for harmonization of categories to allow more accurate interstudy comparisons.

Of note, our findings are in line with similar studies conducted in children with KD, a clinically similar condition. Dionne et al found, in a large retrospective study performed in US between 2000 and 2017, that children with lower socioeconomic status had a greater number of days of fever at time of treatment (*p* = 0.01), longer hospitalizations (*p* = 0.007) and were more likely to develop large/giant CAA (*p* = 0.03).^23^ In England, KD incidence seems to be higher in more deprived socioeconomic groups.^24^ Even in children with acute SARS‐CoV‐2 infection, SDH such as housing instability, food insecurity and higher childcare needs were associated with more severe disease.^25^

Although our study showed a significant correlation between MIS‐C outcomes and SDH, we are not able to explain the reasons for these associations. Some simplistic explanations may be that parents with lower education may not recognize an ill‐child as quickly, or parents having low salaries may need to work several hours per day or do multiple jobs to guarantee basic needs for their families, therefore spending less time with their children and thus recognizing worsening of their clinical conditions later in the course of illness. In addition, some parents or guardians may have a form of employment that does not allow them to take time off to seek care for an ill child.

Independently from the causes, our findings may have implications. Since children with MIS‐C (and KD, as described in other studies) living in more difficult social conditions have worse outcomes, these findings may guide preventive and informative strategies, for example prioritizing poorer areas and high‐risk populations. In fact, a single study we are aware of investigating MIS‐C awareness among US parents found that higher educational level (compared to high school degree; some college: odds ratio [OR], 2.00 [95% confidence interval {CI}, 1.44–2.77]; bachelor's degree or higher: OR, 3.14 [95% CI, 2.26–4.35]) was associated with MIS‐C awareness.^26^

Despite this study being the largest study to investigate the role of social factors on MIS‐C outcomes, these data were available only from a minority of MIS‐C cases included in the REKAMLATINA REDCap. Also, not all Latin American countries were equally represented. Other relevant social factors may have not been considered, for example the presence of active grandparents (and their cultural and economic status) in the family that might support parents caring for children. The social score we used, although adapted from a previous publication, has not been validated by other studies. Also, although the percentage of death in Latin America due to MIS‐C is higher than other parts of the world,^27^, ^28^ we had SDH on only a few children who died and therefore were unable to determine SDH that were associated with the most severe form of MIS‐C. Nevertheless, our findings should inform policy makers to establish specific interventions to reduce those modifiable factors that have the potential to negatively impact health outcomes, particularly in the most fragile areas.

In conclusion, we found that among a large cohort of Latin American children with MIS‐C, SDH like food insecurity, larger distance from a health center, not owning a car to transport the patient to hospital, payment by other means, or having a home in poor condition were associated with decreased left ventricular ejection fraction, shock, transfusion, and need for respiratory support. These findings reinforce the need for better investigation of the role of social determinants of health in MIS‐C and other inflammatory conditions and may guide public health interventions.

## AUTHOR CONTRIBUTIONS

**Danilo Buonsenso:** Conceptualization; investigation; writing—original draft; methodology; validation; writing—review and editing. **Anna Camporesi:** Methodology; data curation; software; formal analysis; writing—original draft. **Charles Sawaya:** Conceptualization; data curation; writing—review and editing. **Rolando Ulloa‐Gutierrez:** Conceptualization; investigation; writing—original draft; writing—review and editing; methodology; project administration; data curation; supervision; resources. **Enrique Faugier‐Fuentes:** Investigation; writing—review and editing; visualization. **Lourdes Dueñas:** Investigation; writing—review and editing; visualization. **Nancy Gálvez‐Rafael:** Investigation; visualization; writing—review and editing. **Andrea Gatica:** Investigation; visualization; writing—review and editing. **Patricia Saltigeral‐Simental:** Investigation; visualization; writing—review and editing. **Adán Cuatecontzi‐Romero:** Investigation; visualization; writing—review and editing. **Flávia Jacqueline Almeida:** Investigation; visualization; writing—review and editing. **Shirley Cuan:** Investigation; visualization; writing—review and editing. **Sandra Beltrán:** Investigation; visualization; writing—review and editing. **Erika Reina‐Bautista:** Investigation; visualization; writing—review and editing. **Adrián Collia:** Investigation; visualization; writing—review and editing. **Gabriela Ivankovich‐Escoto:** Investigation; visualization; writing—review and editing. **Jaime Fernández‐Sarmiento:** Investigation; visualization; writing—review and editing.

## The REKAMLATINA‐3 MIS‐C Study Group Investigators

**Elizabeth Moreno** (University of California, San Diego, CA, USA), **Jimena García‐Silva** Facultad de Medicina, Universidad Autónoma de Nuevo León; Monterrey, México Medical Student, **Issa Lorena López‐Medina** (Clínica Pediátrica Colsanitas, Bogotá, Colombia), **Manuel Munaico, Róger Hernández, Gian Huamán** (Clínica San Felipe; Lima, Perú)), **Maria C Cervi** (Universidade de Sāo Paolo; Sāo Paolo, Brazil), **Alejandro Ellis** (Sanatorio Mater Dei, Buenos Aires, Argentina), **Daniel Jarovsky** (Santa Casa de Sāo Paulo School of Medicinal Sciences; Sāo Paulo, Brazil), **Lorena Franco** (Hospital Infantil Municipal de Córdoba; Córdoba, Argentina), **Kathia Luciani** (Hospital de Especialidades Pediátricas Omar Torrijos Herrera; Ciudad de Panamá, Panamá), **Enrique Chacon‐Cruz** (Hospital General de Tijuana; Tijuana, México & Think Vaccines LLC; Houston, TX, USA), **Alejandro Díaz** (Hospital General de Medellín, Medellín, Colombia), **Maynor G Bravo‐López** (Hospital Herrera Llerandi, Ciudad Guatemala, Guatemala), **Marco A Yamazaki‐Nakashimada, Itzel Ríos‐Olivares, Martha Márquez‐Aguirre** (Instituto Nacional de Pediatría; Ciudad de México, México), **Adriana Yock‐Corrales** (Hospital Nacional de Niños “Dr. Carlos Sáenz Herrera”, Centro de Ciencias Médicas, Caja Costarricense de Seguro Social (CCSS), San José, Costa Rica), **Mónica Pujadas** (Hospital Pediátrico Centro Hospitalario Pereira Rossell, Montevideo, Uruguay), **Ana Victoria Villarreal‐Treviño** (Hospital Regional Materno Infantil de Alta Especialidad; Monterrey, México).

## CONFLICT OF INTEREST STATEMENT

The authors declare no conflict of interest.

## ETHICAL APPROVAL STATEMENT

The study was approved by the REKAMLATINA local ethics committees and informed consent was provided based on local regulations.

## Supporting information

Supporting information.

## Data Availability

The data that support the findings of this study are available on request from the corresponding author. The data are not publicly available due to privacy or ethical restrictions.
