# Supplementary material for: Impact of social determinants of health on the outcomes of Latin American children with Multisystem Inflammatory Syndrome (MIS‐C)
Source: Pediatr Pulmonol. 2024 Dec 26;60(1):e27313. doi: 10.1002/ppul.27313 (PMC11715135; doi:10.1002/ppul.27313)
Supplement: Supplementary file 1 — Supporting information. [file PPUL-60-0-s001.docx]

**Supplementary Material**

**Social determinants of health in the four main countries**

| **Colonna1** | **Total** | **Brazil** | **Guatemala** | **Mexico** | **Salvador** | **p-value** |
| --- | --- | --- | --- | --- | --- | --- |
|  |  |  |  |  |  |  |
|  | N=241 | N=29 | N=38 | N=144 | N=30 |  |
|  |  |  |  |  |  |  |
| Maternal education |  |  |  |  |  | <0.001 |
| Not attended school | 2 (0.8%) | 0 (0.0%) | 0 (0.0%) | 2 (1.4%) | 0 (0.0%) |  |
| Primary school not completed | 11 (4.6%) | 1 (3.4%) | 2 (5.3%) | 2 (1.4%) | 6 (20.0%) |  |
| Primary school completed | 13 (5.4%) | 0 (0.0%) | 1 (2.6%) | 6 (4.2%) | 6 (20.0%) |  |
| Secondary school not completed | 39 (16.2%) | 6 (20.7%) | 4 (10.5%) | 24 (16.7%) | 5 (16.7%) |  |
| Secondary school completed | 94 (39.0%) | 12 (41.4%) | 14 (36.8%) | 57 (39.6%) | 11 (36.7%) |  |
| Techinical school | 31 (12.9%) | 3 (10.3%) | 9 (23.7%) | 17 (11.8%) | 2 (6.7%) |  |
| University | 37 (15.4%) | 3 (10.3%) | 8 (21.1%) | 26 (18.1%) | 0 (0.0%) |  |
| Missing | 14 (5.8%) | 4 (13.8%) | 0 (0.0%) | 10 (6.9%) | 0 (0.0%) |  |
|  |  |  |  |  |  |  |
| Paternal education |  |  |  |  |  | <0.001 |
| Primary school not completed | 6 (2.5%) | 0 (0.0%) | 0 (0.0%) | 2 (1.4%) | 4 (13.3%) |  |
| Primary school completed | 8 (3.3%) | 1 (3.4%) | 0 (0.0%) | 6 (4.2%) | 1 (3.3%) |  |
| Secondary school not completed | 23 (9.5%) | 8 (27.6%) | 3 (7.9%) | 9 (6.2%) | 3 (10.0%) |  |
| Secondary school completed | 75 (31.1%) | 6 (20.7%) | 13 (34.2%) | 49 (34.0%) | 7 (23.3%) |  |
| Techinical school | 32 (13.3%) | 3 (10.3%) | 10 (26.3%) | 12 (8.3%) | 7 (23.3%) |  |
| University | 39 (16.2%) | 0 (0.0%) | 10 (26.3%) | 27 (18.8%) | 2 (6.7%) |  |
| Missing | 58 (24.1%) | 11 (37.9%) | 2 (5.3%) | 39 (27.1%) | 6 (20.0%) |  |
|  |  |  |  |  |  |  |
| Household facilities |  |  |  |  |  |  |
|  |  |  |  |  |  |  |
| Electricity | 236 (97.9%) | 28 (96.6%) | 38 (100.0%) | 140 (97.2%) | 30 (100.0%) | 0.56 |
|  |  |  |  |  |  |  |
| Running water | 204 (84.6%) | 28 (96.6%) | 36 (94.7%) | 122 (84.7%) | 18 (60.0%) | <0.001 |
|  |  |  |  |  |  |  |
| water_hole1 | 21 (8.7%) | 0 (0.0%) | 0 (0.0%) | 9 (6.2%) | 12 (40.0%) | <0.001 |
|  |  |  |  |  |  |  |
| Sanitary water | 198 (82.2%) | 28 (96.6%) | 38 (100.0%) | 115 (79.9%) | 17 (56.7%) | <0.001 |
|  |  |  |  |  |  |  |
| Latrine | 31 (12.9%) | 0 (0.0%) | 2 (5.3%) | 17 (11.8%) | 12 (40.0%) | <0.001 |
|  |  |  |  |  |  |  |
| Collection of solid waste | 173 (71.8%) | 24 (82.8%) | 36 (94.7%) | 113 (78.5%) | 0 (0.0%) | <0.001 |
|  |  |  |  |  |  |  |
| Payment of hospital |  |  |  |  |  |  |
|  |  |  |  |  |  |  |
| Public insurance | 88 (36.5%) | 28 (96.6%) | 13 (34.2%) | 45 (31.2%) | 2 (6.7%) | <0.001 |
|  |  |  |  |  |  |  |
| Private payment | 19 (7.9%) | 2 (6.9%) | 12 (31.6%) | 5 (3.5%) | 0 (0.0%) | <0.001 |
|  |  |  |  |  |  |  |
| Sell payment | 23 (9.5%) | 0 (0.0%) | 1 (2.6%) | 22 (15.3%) | 0 (0.0%) | 0.003 |
|  |  |  |  |  |  |  |
| Savings payment | 50 (20.7%) | 1 (3.4%) | 15 (39.5%) | 34 (23.6%) | 0 (0.0%) | <0.001 |
|  |  |  |  |  |  |  |
| Other payment | 33 (13.7%) | 0 (0.0%) | 4 (10.5%) | 1 (0.7%) | 28 (93.3%) | <0.001 |
|  |  |  |  |  |  |  |
| Loan payment | 55 (22.8%) | 2 (6.9%) | 5 (13.2%) | 48 (33.3%) | 0 (0.0%) | <0.001 |

| **Colonna1** | **Total** | **Brazil** | **Guatemala** | **Mexico** | **Salvador** | **p-value** |
| --- | --- | --- | --- | --- | --- | --- |
|  |  |  |  |  |  |  |
|  | N=241 | N=29 | N=38 | N=144 | N=30 |  |
|  |  |  |  |  |  |  |
| Presence of non-relatives | |  |  |  |  | 0.063 |
| 0 | 215 (89.2%) | 27 (93.1%) | 37 (97.4%) | 129 (89.6%) | 22 (73.3%) |  |
| 1 | 16 (6.6%) | 1 (3.4%) | 1 (2.6%) | 8 (5.6%) | 6 (20.0%) |  |
| 2 | 2 (0.8%) | 0 (0.0%) | 0 (0.0%) | 1 (0.7%) | 1 (3.3%) |  |
| 3 | 2 (0.8%) | 0 (0.0%) | 0 (0.0%) | 2 (1.4%) | 0 (0.0%) |  |
| 5 | 1 (0.4%) | 0 (0.0%) | 0 (0.0%) | 0 (0.0%) | 1 (3.3%) |  |
| Missing | 5 (2.1%) | 1 (3.4%) | 0 (0.0%) | 4 (2.8%) | 0 (0.0%) |  |
|  |  |  |  |  |  |  |
| Presence of people aged 65 or more | |  |  |  |  | <0.001 |
| 0 | 168 (69.7%) | 25 (86.2%) | 35 (92.1%) | 97 (67.4%) | 11 (36.7%) |  |
| 1 | 42 (17.4%) | 3 (10.3%) | 0 (0.0%) | 23 (16.0%) | 16 (53.3%) |  |
| 2 | 23 (9.5%) | 0 (0.0%) | 3 (7.9%) | 17 (11.8%) | 3 (10.0%) |  |
| 3 | 1 (0.4%) | 0 (0.0%) | 0 (0.0%) | 1 (0.7%) | 0 (0.0%) |  |
| Missing | 7 (2.9%) | 1 (3.4%) | 0 (0.0%) | 6 (4.2%) | 0 (0.0%) |  |
|  |  |  |  |  |  |  |
| Presence of persons with disability | |  |  |  |  | 0.073 |
| 0 | 213 (88.4%) | 28 (96.6%) | 37 (97.4%) | 118 (81.9%) | 30 (100.0%) |  |
| 1 | 19 (7.9%) | 0 (0.0%) | 1 (2.6%) | 18 (12.5%) | 0 (0.0%) |  |
| 2 | 2 (0.8%) | 0 (0.0%) | 0 (0.0%) | 2 (1.4%) | 0 (0.0%) |  |
| Missing | 7 (2.9%) | 1 (3.4%) | 0 (0.0%) | 6 (4.2%) | 0 (0.0%) |  |
|  |  |  |  |  |  |  |
| Presence of children under 5 | |  |  |  |  | 0.54 |
| 0 | 129 (53.5%) | 16 (55.2%) | 20 (52.6%) | 73 (50.7%) | 20 (66.7%) |  |
| 1 | 87 (36.1%) | 12 (41.4%) | 13 (34.2%) | 53 (36.8%) | 9 (30.0%) |  |
| 2 | 13 (5.4%) | 0 (0.0%) | 5 (13.2%) | 7 (4.9%) | 1 (3.3%) |  |
| 3 | 2 (0.8%) | 0 (0.0%) | 0 (0.0%) | 2 (1.4%) | 0 (0.0%) |  |
| 4 | 1 (0.4%) | 0 (0.0%) | 0 (0.0%) | 1 (0.7%) | 0 (0.0%) |  |
| Missing | 9 (3.7%) | 1 (3.4%) | 0 (0.0%) | 8 (5.6%) | 0 (0.0%) |  |
|  |  |  |  |  |  |  |
| Presence of children 5-11 y | |  |  |  |  | 0.26 |
| 0 | 128 (53.1%) | 17 (58.6%) | 24 (63.2%) | 67 (46.5%) | 20 (66.7%) |  |
| 1 | 86 (35.7%) | 8 (27.6%) | 13 (34.2%) | 56 (38.9%) | 9 (30.0%) |  |
| 2 | 17 (7.1%) | 2 (6.9%) | 1 (2.6%) | 13 (9.0%) | 1 (3.3%) |  |
| 3 | 1 (0.4%) | 1 (3.4%) | 0 (0.0%) | 0 (0.0%) | 0 (0.0%) |  |
| 4 | 1 (0.4%) | 0 (0.0%) | 0 (0.0%) | 1 (0.7%) | 0 (0.0%) |  |
| Missing | 8 (3.3%) | 1 (3.4%) | 0 (0.0%) | 7 (4.9%) | 0 (0.0%) |  |
|  |  |  |  |  |  |  |
| Presence of children of children >11 y | |  |  |  |  | 0.028 |
| 0 | 174 (72.2%) | 20 (69.0%) | 32 (84.2%) | 94 (65.3%) | 28 (93.3%) |  |
| 1 | 34 (14.1%) | 5 (17.2%) | 1 (2.6%) | 26 (18.1%) | 2 (6.7%) |  |
| 2 | 20 (8.3%) | 1 (3.4%) | 4 (10.5%) | 15 (10.4%) | 0 (0.0%) |  |
| 3 | 2 (0.8%) | 0 (0.0%) | 1 (2.6%) | 1 (0.7%) | 0 (0.0%) |  |
| 4 | 1 (0.4%) | 1 (3.4%) | 0 (0.0%) | 0 (0.0%) | 0 (0.0%) |  |
| 5 | 1 (0.4%) | 1 (3.4%) | 0 (0.0%) | 0 (0.0%) | 0 (0.0%) |  |
| 90 | 1 (0.4%) | 0 (0.0%) | 0 (0.0%) | 1 (0.7%) | 0 (0.0%) |  |
| Missing | 8 (3.3%) | 1 (3.4%) | 0 (0.0%) | 7 (4.9%) | 0 (0.0%) |  |
|  |  |  |  |  |  |  |
| Presnece of children with disability | |  |  |  |  | 0.2 |
| 0 | 226 (93.8%) | 28 (96.6%) | 38 (100.0%) | 130 (90.3%) | 30 (100.0%) |  |
| 1 | 7 (2.9%) | 0 (0.0%) | 0 (0.0%) | 7 (4.9%) | 0 (0.0%) |  |
| Missing | 8 (3.3%) | 1 (3.4%) | 0 (0.0%) | 7 (4.9%) | 0 (0.0%) |  |
|  |  |  |  |  |  |  |
| Number of rooms in the household | 2.0 (2.0-3.0) | 2.0 (2.0-2.5) | 4.0 (3.0-5.0) | 2.0 (2.0-3.0) | 2.0 (2.0-2.0) | <0.001 |
|  |  |  |  |  |  |  |
| Number of persosn living iin the households | 1.7 (1.2-2.3) | 1.5 (1.0-2.0) | 1.0 (0.6-1.4) | 2.0 (1.5-2.5) | 1.7 (1.5-2.0) | <0.001 |
